# Editorial to “Association between ventricular arrhythmia (premature ventricular contractions burden and non‐sustained ventricular tachycardia) and cardiovascular events in patients without structural heart disease”

**DOI:** 10.1002/joa3.13219

**Published:** 2025-01-20

**Authors:** Wei Sheng Jonathan Ong, Chi Keong Ching

**Affiliations:** ^1^ Department of Cardiology National Heart Centre Singapore Singapore Singapore

**Keywords:** cardiovascular events, heart failure, non‐sustained ventricular tachycardia, premature ventricular contractions, tachycardia‐induced cardiomyopathy

Whether frequent premature ventricular contractions (PVCs) in patients without structural heart disease are of prognostic significance is a subject of debate.^1^ Once considered to be a benign condition, it is now widely known that it can be causative for tachycardia‐induced cardiomyopathy. While only a minority of patients with frequent PVCs (>1000 PVCs/day) develop ventricular dysfunction after 5 years of follow‐up,^2^ catheter ablation is curative for these patients with normalization of cardiac function. The minimal threshold for the development of LV dysfunction is a PVC burden of 10% while a PVC burden of >20% portends a higher risk. Upfront catheter ablation is also indicated in symptomatic patients without structural heart disease when the PVCs are of right ventricular outflow tract or fascicular origin.^3^ Beyond the above select patient groups, however, it remains unclear whether frequent PVCs are associated with cardiovascular events in patients without structural heart disease.

In this issue of the *Journal of Arrhythmia*, Ogiso et al. conducted a single‐center retrospective study with 6332 patients, stratified by the number of baseline PVCs and the presence or absence of non‐sustained ventricular tachycardia (NSVT). The primary endpoint was defined as the incidence of cardiovascular events, including all‐cause death, acute coronary syndrome, ischemic stroke, systemic embolism, and hospitalization for heart failure. The authors reported that, over a 3 year follow‐up period, the frequency of PVCs was not associated with cardiovascular events while the presence of NSVT was associated with a higher risk of heart failure hospitalization. In the NSVT study population, only one of the five cases of heart failure had a reduced ejection fraction.

Notably, these results differ from previous studies^4^, ^5^; however, this can be explained on more careful examination of key study differences. Prior studies have shown that the decrease in cardiac function, increase in heart failure events, and mortality among patients with frequent PVCs were normally noted beyond 5 years of follow‐up.^4^, ^5^ This suggests that the 3 year follow‐up period in the study may have been inadequate to detect these differences. Furthermore, as pointed out by the authors, increased use of medical interventions such as anti‐arrhythmic drugs and catheter ablation in patients with a larger number of PVCs and NSVT may have contributed to a better prognosis and outcome.

Ogiso et al. reported that one patient with NSVT and heart failure was later diagnosed with hypertrophic cardiomyopathy. This was not detected at baseline with echocardiography. As frequent PVCs and NSVT may indicate subclinical abnormalities, the authors opined that further investigations, including cardiac magnetic resonance imaging (MRI), may be needed in select patients. This recommendation is in line with the ESC guidelines, which recommend cardiac MRI in patients with inconclusive prior investigations or who have an atypical presentation (older age, right bundle branch block morphology).^3^ The importance of a thorough diagnostic workup for structural heart disease in patients with frequent PVCs and NSVT cannot be overstated as it can significantly alter management with consequent downstream effects on patient prognosis and outcome.

Finally, limitations in the current study design must be recognized. This was a single‐center study conducted in a hospital, which exclusively specializes in cardiovascular medicine. As such, its patient population may have achieved stricter control of their cardiovascular risk factors, were initiated on anti‐arrhythmic therapy more readily and sent for catheter ablation expediently when indicated. This is not analogous to the general community population making the study results difficult to apply to the general population at large.

Many questions still remain pertaining to any association between PVCs and NSVT and cardiovascular events in patients without structural heart disease. Additional data, such as the origin of the PVCs and NSVT and PVC morphology, would further enrich the database and enhance our understanding. Likewise, long‐term outcome data extending beyond 5 years may offer a clearer insight into any potential association. A multi‐center study would also help to strengthen research findings. Future research incorporating these elements will help to fill existing knowledge gaps. Nevertheless, studies of this nature are limited and rare, and this study contributes valuable insights to the field.

## CONFLICT OF INTEREST STATEMENT

Authors declare no conflict of interests for this article.
